# What Fate for Plastics in Artworks? An Overview of Their Identification and Degradative Behaviour

**DOI:** 10.3390/polym13060883

**Published:** 2021-03-13

**Authors:** Massimo Lazzari, Daniela Reggio

**Affiliations:** 1 Departamento de Química Física, Facultade de Química, Universidade de Santiago de Compostela, 15701 Santiago de Compostela, Spain; daniela.reggio@usc.es; 2 Centro Singular de Investigación en Química Biolóxica e Materiais Moleculares (CiQUS), Universidade de Santiago de Compostela, 15705 Santiago de Compostela, Spain

**Keywords:** polymer oxidation, durability, polymer ageing, contemporary art, cultural heritage, plasticisers, FTIR spectroscopy, py-GC/MS, NMR spectroscopy, handheld techniques

## Abstract

This review is conceived as a guide for material science researchers and conservators aiming to face the problem of deterioration of contemporary artworks entirely or partially made of plastics. It initially illustrates the analytical approaches for identifying polymeric material components in 3D art objects, such as sculptures and installations, and provides a perspective of their limits and advantages. Subsequently, the methodologies used for studying the deterioration of contemporary art plastics are reviewed, emphasising the main effects of the different types of degradation (i.e., migration of additives, oxidation and hydrolysis) and suggesting the appropriate techniques for their detection. Finally, the application of artificial ageing tests is critically assessed. All the concepts are elaborated through case studies and examples.

## 1. Introduction

Artists and designers have used materials produced for industrial purposes since the last part of the nineteenth century [1]. Especially from the 1930s, advances in macromolecular chemistry have provided a variety of materials with new properties and relatively low costs, giving opportunities for creative experimentations that have led to the gradual substitution of natural materials with synthetic ones [2]. Degradative mechanisms and conservation treatments of traditional art supplies are relatively well established. In contrast, there is limited research providing sound evidence-based methodologies for researching modern and contemporary art polymers. Ageing of synthetic, semi-synthetic and natural polymers has been extensively studied, as well as the improvement of short- and medium-term performances [3]. However, if we only refer to plastics, here intended as polymer-based materials which can be manipulated to produce art objects, such as sculptures or installations, only some, often unrelated, degradation studies have focused on their long-term durability. Hopefully, searching the available data and summarising them as a tool for chemists, conservators and material scientists interested in putting synthetic polymers into a heritage science perspective might be of use to foster research and fill existing knowledge gaps. On the other side, plastic-containing artworks are among the few examples of plastic artefacts that have been conceived to have a long lifespan. Some artworks already aged for more than 50 years and their study offer first-hand information on industrial plastics’ durability.

Concerning a more general presence of polymers in cultural heritage materials, polymeric components have been added to modern paints as binders since the 1930s and several studies have addressed their long-term stability [4,5,6,7,8]. Many synthetic materials are used as coatings and consolidants for the conservation of heritage surfaces [9,10,11,12,13]. Moreover, contemporary collections have large amounts of design objects, furniture and toys constituted by early plastics decaying visibly in museum environments [14,15,16,17]. Museums condition surveys report that 1% of objects in plastic collections are affected by severe deterioration, and 12% show visible signs of decay [17]. Further, over 700 plastic objects in the Victoria and Albert Museum and the British Museum revealed that 5–25 years after production, they start displaying ageing signs, such as colour change and odour emissions. Shrinkage and stickiness are other signs of degradation recorded [18].

In addition, plastics’ durability is an especially relevant requisite for collectability. To date, the majority of plastics collected, and hence potentially researched by material scientists, are design objects; however, knowledge advancement about objects with a greater artistic and economic value, such as sculptures, could have a higher impact on current societal concerns [19]. First, the object has to be rare, which contradicts the mass production of plastic. Second, the impactful design, the use of colour, the technical precision and the variety of shapes and functions should be unique. Third, the object’s artistic components and elegance and its resulting innovations make it more desirable for a collection [20].

Private and public collectors keep investing a considerable amount of money in contemporary art objects, and undoubtedly synthetic materials have a prominent role in the fabrication of such works. Artprice.com’s most recent report shows that in the last 20 years, the international market of contemporary art had an average growth of 1370% [21].

Contemporary art is among the healthiest sectors of the modern economy, and even in times of global financial crisis, it has shown exponential growth. Therefore, heritage scientists are urged to fully understand synthetic polymers’ long-term behaviours in artworks to respond to inevitable upcoming losses, and upgrade their knowledge on emerging materials (e.g., 3D printing polymers) [22]. The report also confirms that “masterpieces”, i.e., monumental works fabricated with more durable supports, have higher returns on sales (e.g., return of + 1000% from Basquiat’s canvases over + 4% on his printed drawings sold in the last 17 years). Regardless of stability, most artists’ choices respond to aesthetic needs, rather than functional ones. As an example, Newman discusses plastics as the new art medium, and thoroughly and chronologically lists polymers, their workability and their aesthetic properties [23].

In this review, the first research question relates to the analytical techniques used to identify and assess the durability of polymer components in artworks. The second research question aims at evaluating whether artificial ageing tests are the most efficient investigation method to predict artwork durability. Its overall aim is to critically present and discuss all the scientific literature about polymer analysis and degradation from a conservation science perspective but also be of general interest for a polymer scientist readership. The objectives are: (i) to summarise previous research about synthetic polymers used for the fabrication of contemporary art objects; (ii) to compare and contrast data-based research about polymer degradation behaviours; (iii) select the best methodologies of analysis and characterisation, and suggest further improvements.

## 2. Identification and Compositional Analysis of Plastics in Artworks

Trademarks, registration marks, and patent numbers are the preliminary data to collect on plastic objects. However, they are seldom available for artworks, apart from the cases in which artists used plastic objects instead of raw materials. On the other hand, visual features as transparency/opacity might be of aid in sorting plastics. Odour is also considered a preliminary tool for polymer identification if materials are already showing extensive deterioration [17,19,24,25]. Destructive, non-instrumental identification techniques, such as those based on hardness, density and hot pin tests were historically used to analyse polymers in collections but are only exceptionally applied on artwork materials and will not be discussed further here. To give a first overview of the large variety of polymers used in artworks, Table 1 presents a non-exhaustive list with artists.

Instrumental analysis allows identifying and characterising the different plastic components in artefacts, comprising polymeric blends, and additives (including dyes and pigments). Further, additives may migrate from the polymeric material and degrade at different rates compared to bulk polymers, and being able to identify and quantify them has proven to be extremely informative [26]. Molecular weight distributions and polymer microstructure affect physical properties and also plastic stability and degradability; therefore, quantitative analyses of such features can lead to more informed preservation strategies in collections [27,28]. By extension, the same methodologies critically discussed in this review and general considerations may be applied to other museum plastic-containing objects.

Vibrational spectroscopies, especially infrared spectroscopy, are the techniques of choice for polymer structure determination [29]. Differently from the case of old master paintings and sculptures where the use of non-destructive analytical techniques is almost compulsory, contemporary artworks may sometime be investigated by micro-destructive techniques, as fragments weighing a few milligrams can be sampled from the original artworks or collected in the form of debris from artwork storage cases in museums [27,30]. In addition, the availability of the original materials used for their creation in artists’ studios can be a valuable help to facilitate compositional analysis [30]. Further advantage of studying contemporary art materials is that often the artists, or their assistants, are alive and can contribute to documenting the materials through interviews [28,30,31,32,33]. In parallel, to clarify the materials and fabrication methods of contemporary art, a few museum research programs collaborate with artists to create databases of materials. Among dedicated reference spectral libraries devoted to cultural heritage studies, the Sample Collection (SamCo) database, implemented within the POPART European funded project, contains analytical data on natural, semi-synthetic and synthetic plastics used in museum objects, mainly from Fourier transform infrared (FTIR) but also near-infrared (NIR) spectroscopy, pyrolysis-gas chromatography/mass spectrometry (py-GC/MS), evolved gas analysis-mass spectrometry (EGA-MS), Raman spectroscopy and differential scanning calorimetry (DSC) [14,34]. SamCo derives from the previously available ResinKit™ and comprises 100 samples of unaged and naturally aged industrial plastics, as standards and objects, respectively.

More specifically, attenuated total reflectance (ATR) FTIR spectroscopy has successfully identified plastics and additives in collections since it provides reliable results with limited sampling. The use of polymers with common and well-defined structures, mostly vinyl polymers, has been unequivocally revealed, e.g., in the case of PMMA-made vacuum plastics by C. Kauffman [35], in the PVC-packed Christo armchair or the polyethylene gauze of “Citta Irreale” by M. Merz [36], the PI used by A. Pinal for his tailored latex textiles [30] or the variety of polymers, namely, PE, PP, PS and poly(ethylene terephthalate) (PET), found in the sculptures put together by T. Cragg from scavenged plastic objects [37]. Similarly, but to differentiate between the synthetic polymers used in former restorations of works of art and the original materials used by the artist, Domenech-Carbó et al. [38] proposed quantitative diagnostic criteria based on two-dimensional diagrams of the relative intensity of selected FTIR peaks. For example, an accurate assignment of peaks allowed them to identify recently applied acrylics, alkyd resins, cellulose derivatives, poly(vinyl acetate) (PVAc) and semi-synthetic wax in artistic objects.

**Figure 1:**
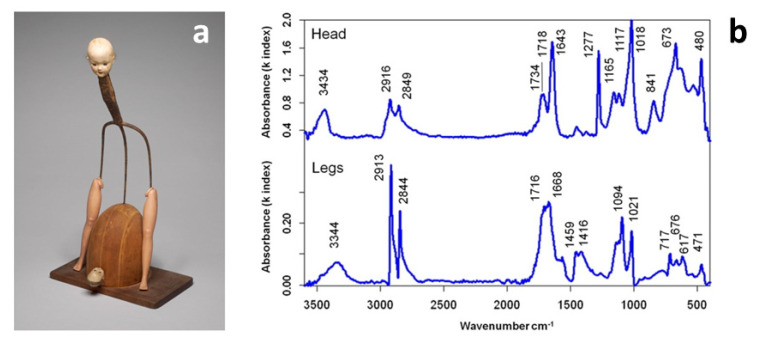
“Pitchfork Lady” (1964) by Don Baum (**a**) and corresponding Kramers-Kronig transformation (KKT) calculated FTIR spectra for the head and the legs (**b**). Image reprinted with permission from [41].

The recent availability of relatively inexpensive portable spectroscopy systems has also paved the way to fast and reliable identification of plastic components in museum collections, with particular attention on the systems that differ from those based on ATR-FTIR which do not require contact of the crystal with the object, exposing the artwork to the risk of visible indentation. Portable ATR coupled with NIR spectroscopy has been used for the identification of reference historical polymers [39], but it was only the use of FTIR reflectance spectroscopy in the mid-infrared range that offered the advantage of portability while enabling non-invasive and contactless analysis [40]. Saviello et al. demonstrated that such in situ analysis successfully identifies plastics and additives through good quality spectra irrespective of the object’s shape or colour. In contrast, severe limitations were observed in thin transparent films and foams that remain extremely challenging to characterise with this technique [41]. Kramers-Kronig transformation (KKT) to correct spectral distortions of reflection spectra was applicable in most cases, and occasional band shifts did not significantly impair the positive identification of the base polymer or blend. Analysis of a complex artwork by Don Baum (Figure 1) allowed for identifying cellulose nitrate (CN) and PE as head and leg constituent materials, respectively, by direct comparison with reference spectra. Additional peaks at ca. 1020 and 675 cm^−1^ were ascribed to talc, whereas the presence of C=O stretching bands (1710–1740 cm^−1^) could indicate the presence of plasticisers or the occurrence of products of polymer degradation, as discussed in the next review section.

FTIR spectroscopy also offers invaluable information for identifying many condensation polymers through the recognition of the main functional groups. PUR, ER, UP and PA are sometimes challenging to unequivocally identify even by using comprehensive databases and applying a complementary analytical technique, such as Raman spectroscopy, py-GC/MS or nuclear magnetic resonance (NMR) spectroscopy may become necessary. That is, without forgetting that any of these techniques may be, and have indeed been, used alone for direct compositional analysis.

**Figure 2:**
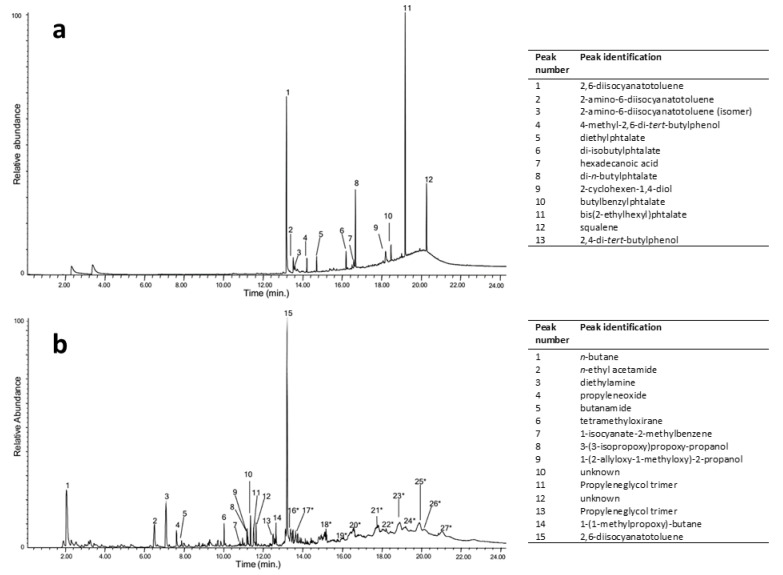
Pyrolysis-GC/MS (Py-GC/MS) chromatograms after first-shot pyrolysis at 306 °C (**a**) and after second-shot pyrolysis at 600 °C (**b**) of a bulk poly(ether urethane) from “Contenitoreumano”, and identification of corresponding chromatographic peaks. Peaks from 16 to 27 correspond to oligomers. Image reprinted with permission from [44].

Due to their lightness and softness, PUR foams have attracted artists and designers since the 1960s. The fact that the two families of foams, i.e., poly(ester urethane) and poly(ether urethane), show different degradative pathways and a wide variety of possible compositions within each family makes their analysis a critical conservation issue. Although FTIR analysis easily detects the family of the PUR used in many contemporary artworks through the presence of characteristic main ester or ether peaks, at around 1120–1150 cm^−1^ (C–O–CO stretching of esters) [42] or 1050–1100 cm^−1^ (C–O–C stretching of polyether polyol) [30,43], respectively, only py-GC/MS or NMR may disclose their composition. The exact nature of a poly(ether urethane) foam used by I. Parisi and F. Somaini for the prototype “Contenitoreumano” has been elegantly proved through double-shot py-GC/MS experiments, carried out at around 300 and 600 °C to collect and partially separate all the developed products (Figure 2) [44]. In the first shot, the most abundant species are free 2,6-diisocyanatetoluene (peak n. 1 in Figure 2a, identified as one of the precursors used in the synthesis of the polyurethane) and related derivates (n. 2,3) and phthalates (n. 5, 6, 8, 10, 11) used as plasticisers. At the higher temperature shot (Figure 2b), the pyrogram is more complex and contains the following information: up to 10 min, peaks refer to the main pyrolysis products derived from chain extenders, between 10 and 13 min mainly to the 2,6-diisocyanatotoluene precursor (n. 15) and rearranged molecules, and different chain length ether oligomers with propyl ether units were observed in the time range of 13–22 min, thus indicating that polypropylene glycol was the polyol used in the synthesis of this PUR foam.

The formulation components of a reference poly(ester urethane) foam used for artefacts could also be revealed by a combination of FTIR and (py-)GC/MS, and confirmed by proton magic angle spinning (^1^H MAS) NMR [42]. The py-GC/MS analysis established the isocyanate as a mixture of 2,4- and 2,6-diisocyanatetoluene. To identify the polyol fraction as polydiethylene glycol adipate, the foam’s extraction in deuterated water was obtained and analysed by GC/MS. Another excellent example of characterisation of the same type of polymer concerns the detection of the constituent material of a contemporary artwork (“Moon Surface”, 1964 by G. Turcato) through the application of different NMR methodologies [45]. Solid state NMR, i.e., carbon cross-polarisation (CP)MAS NMR, allowed the identification of carbonyl carbon of urethane groups. In contrast, the resonances of methyl, methine and methylene carbons were diagnostic of the presence of poly(propylene oxide), possibly capped with poly(ethylene oxide), constituting the soft phase of the PUR foam. More complex 2D analysis, such as solid state ^1^H-^13^C correlation experiments or total correlation spectroscopy (TOCSY) and heteronuclear multiple quantum coherence (HMQC) maps from solution, indicated the presence of diisocyanatotoluene constituting the hard phase of the PUR, and clarified the detailed microstructure of the poly(propylene oxide)-poly(ethylene oxide) copolymer.

Other materials with a wide compositional diversity are UP resins, used in contemporary art and design, because of their lightness and low cost. UP resins are prepared from a saturated dicarboxylic acid or its anhydride (usually phthalic anhydride) and an unsaturated dicarboxylic acid or anhydride (usually maleic anhydride), which are reacted with one or more dialcohols, such as ethylene glycol or propylene glycol. Since the 1960s, it has been popular to use such resins to produce fibreglass-reinforced polymer composite artworks with a three-dimensional shape which often suffer problems of degradability, strictly related to chemical composition and manufacturing process of the polymer composite. An investigation preliminary to conservation treatment of a floating sculpture by M. Pan (“Sculpture flottante, Otterlo”, 1960–61) very efficiently showed that the cured resin was pyrolysed into styrene, phthalic acid, short glycols and cinnamic acid. Most compounds relate to the original building blocks of the polymer, with styrene being the vinyl monomer used for the copolymerisation, and the cinnamic acid the linkage between the reactive double bonds (fumaric acid) and styrene [46]. In other studies, a combination of FTIR spectroscopy with X-ray diffractometry, and 1D and 2D NMR analysis, revealed the composition and multilayer structure of painted fibreglass-reinforced structures by A. Sassu (“Nuredduna”, 1995) [47] and other artists (“The Last Milk Platform”, 1992, by J.-E. Andersson and “Cocotte with Two Dogs”, 1987, by K. Tykkylainen) [48].

Concerning PA, different artists have occasionally used these polymers since the 1960s, with a special mention for the early linear constructions by N. Gabo, and may be easily recognised by FTIR spectroscopy. Nevertheless, identifying their exact nature, which strongly influences their degradability and long-term behaviour, may require further characterisation through non-spectroscopic techniques. As examples, the linen used in Gabo’s sculpture showed DSC curves with the typical features of polyamide 6,6 [49], and also the industrial PA used for a plastic-made installation by Esferobite DSk could be identified through the DSC measurement of its temperature of fusion, i.e., 173 °C, unequivocally assigned to PA12 [50].

It is also worth citing a preliminary investigation by Necemer et al. aiming to access the capabilities of energy dispersive X-ray fluorescence spectrometry (EDXRF) with monochromatic excitation for the characterisation of the plastic materials used in artefacts from museum collections, e.g., PE, PP, PMMA, PET and PVC [51]. A nondestructive analytical protocol was developed on the basis of the intensity of the coherent and the incoherent scattered excitation radiation from artefacts, compared with scattering from reference plastic materials. A further improvement of the applicability is expected with the diffusion of handheld EDXRF spectrometers, especially in in situ analysis in museums and galleries.

Finally, Table 2 summarises the most reliable techniques used to date for the identification of plastics in artworks. It is not exhaustive but may be considered as a first recommendation to address polymeric component analysis in museum practice.

## 3. Methodological Approaches to the Study of Deterioration

The study of degradation mechanisms and stabilisation processes has been fundamental, since the Second World War with the introduction of new classes of synthetic polymers, and later in the 1950s and 1960s, when polymers became omnipresent in our daily life, in defining polymer uses and industrial applications. Researchers interested in fundamental properties of polymeric materials such as ageing performance or mechanical properties, including dimensional stability and shrinkage, may refer to any polymer encyclopedia or reference book on polymer degradation and stability [3]. In addition, typical damages in museum plastic objects considered as case studies are shown in an atlas released at the end of the POPART project [34] and in conservation-focused books [17,19].

On the other hand, the interest in issues relating to the durability of modern and contemporary artworks, entirely or partially realised in synthetic polymers, is relatively recent. Only in recent decades have museums, conservators and heritage scientists focused their attention on the extent of the degradation of plastics in collections and the factors causing their breakdown [17,30]. It is assumed that artists moved to commercial materials without considering that they were developed for applications and purposes far different from those considered essential from an artistic point of view, where aesthetic rendering is much more important than, e.g., the durability of artworks. The fact that polymers used in contemporary art sculptures often face serious degradation problems, consisting of slight colour changes, cracking, warping, crazing, delaminating, liquid and solid surface exudation or crumbling, is considered to be strictly related to the following two main circumstances. First, the selection by the artist of materials developed for short- or medium-term household and industrial applications, second, the inappropriate manipulation or preparation of the polymeric materials, especially in the case of thermosetting resins or composites, which introduce further factors of untimely degradation [27].

Physical factors of polymer deterioration that are especially important in heritage collections include migration of additives (plasticisers, lubricants, stabilisers, etc.) and interaction with the surroundings, particularly with light and oxygen, in some cases quickened by mechanical stress due to specific uses or handling. Before moving on to reviewing the analytical methodology applied for studying any of these different factors, it is essential to stress the following, often misunderstood, points: (i) the problem of additive migration is particularly important only for objects/artworks made of/containing some specific polymer types, e.g., plasticised PVC or plasticised elastomers, where the plasticiser content may be relevant; (ii) ageing by oxidation, accelerated by the effect of light, heat, mechanical stress or secondary components is ubiquitous and occurs in all the organic materials, as well as under the mild and controlled conditions found in museums and art collections; (iii) the long-term process of elimination of small molecules from polymeric structures such as those of PVC and PVAc, i.e., by dehydrochlorination and deacetylation, respectively, forming chromophores, occurs spontaneously even at room temperature but is very structurally specific.

### 3.1. Migration of Additives

A slow release over time of plasticisers from polymer bulk, such as the typical phthalates extensively used in PVC and many other polymers, causes the exudation of a sticky and oily film onto the artefact surface before complete evaporation takes place. Moisture and dust also accumulate on the films and may initiate further degradative processes. Other examples of migration are those related to the presence of lubricants, such as stearic acid. It is used up to 3% by weight in PVC and other polymers, and yellows and separates from the polymer, causing embrittlement of the plastic surface with time. Further, blooming is caused by salt residues derived from the exudation of flame retardants and those plasticisers that turn solid at room temperature [17].

To extend the lifespan of plasticised polymers used in a variety of industrial products, extensive experimental research has been carried out to understand their thermal or photochemical degradation [52,53], often using strong artificial ageing tests. Less attention has been paid to artificial thermal ageing at moderate temperatures and relatively high relative humidity that could represent the museum environment. In such a sense, the investigation by Royaux at al., aiming to assess poly(ester urethane) preservation strategies for plasticised PVC-based heritage objects, is particularly relevant [54]. Several polymer characterisation techniques were applied to deepen the comprehension of plasticised PVC ageing at both macroscopic and molecular levels, including a better understanding of the impact of conditioning and the effect of cleaning on further plasticiser migration. Although plasticised PVC is not commonly used in artworks, this study may be considered an example of the methodology to be applied for studying the behaviour of artworks realised with additive-containing plastics.

**Figure 3:**
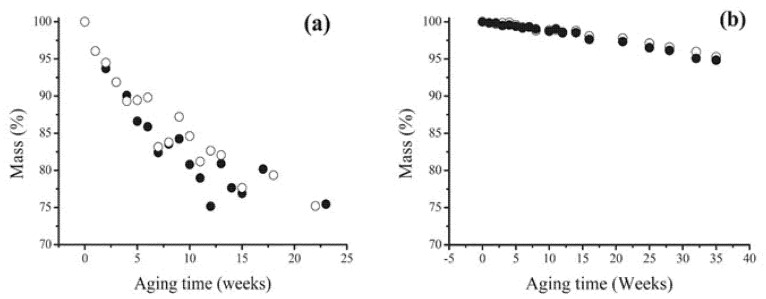
Mass vs. artificial ageing time for as-collected (○) and cleaned (●) 30-year-old PVC films in open (**a**) and closed (**b**) environments. Image reprinted with permission from [54].

**Figure 4:**
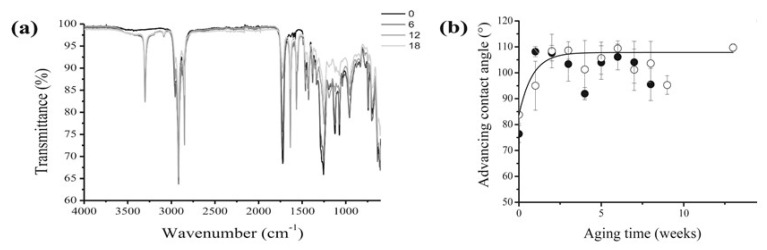
ATR-FTIR spectra measured for cleaned 30-year-old PVC at different durations (in weeks) of artificial thermal ageing (**a**) and evolution of the advancing contact angle as a function of the artificial ageing time for an as-collected (○) and a cleaned (●) 30-year-old PVC film in an open environment (**b**). Image reprinted with permission from [54].

Plasticised PVC films aged for about 30 years in museum conditions, and already showing surface exudates, were submitted to an artificial ageing test consisting in a temperature cycle (2 days at 80 °C then 1 day at 25 °C) under a relative humidity of 65%, and the physico-chemical changes, examined by weighing, FTIR and UV visible spectroscopies, GC/MS, dynamic mechanical analysis, contact angle measurements and other techniques. The effect of a preliminary mechanical surface cleaning was also studied. As expected, samples either freely hung or placed in a closed glass vessel displayed both a progressive loss of plasticiser (Figure 3), and a corresponding loss of flexibility. Such a trend was significantly retarded in closed environment ageing, but almost unaffected by preliminary cleaning. From a methodological point of view, handy information could be collected from the combination of ATR-FTIR analysis with advancing contact angle measurements (Figure 4), which suggested crystalline blooming over the PVC surface of a further, hydrophobic, processing additive identified as a protein, possibly zein. The 3303 cm^−1^ peak is characteristic of N-H stretching modes of a secondary amine while the bands at 1565 cm^−1^ could correspond to the N-H deformation mode and the 1636 cm^−1^ band to the carbonyl group of an amide. The advanced contact angle increases from 84 °C for the as-collected PVC to around 107 °C and simply emphasises the additive migration.

Although further studies are necessary to extend the conclusions to other plasticised polymers, the study by Royaux et al. concludes that, at least for PVC, an efficient preservation strategy may pursue a longer conservation of properties, including visual appearance, while maintaining the artefacts in closed containers. The results also provide crucial information on applying treatments, removing additive surface accumulation to improve their visual aspect without any subsequent degradation.

**Figure 5:**
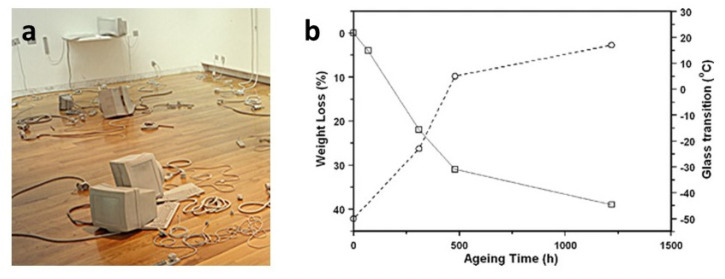
Partial view of “Stage Evidence” (1999) by L. Cecchini (**a**) and weight loss (solid line) and glass-transition temperature (dashed line) of the poly(ether urethane) used in the artwork as a function of the isothermal treatment duration at 120 °C (**b**). Image reprinted with permission from [30].

Another example of migration of plasticisers in contemporary artworks has been revealed in an installation by L. Cecchini, composed of many rubber objects. GC/MS identified the yellowish sticky liquid exuded from their surface as a mixture of phthalates contained in one of the components used to prepare the poly(ether urethane) elastomer, identified by FTIR spectroscopy [30]. An artificial ageing test under isothermal conditions allowed the acceleration of the removal of plasticisers, as examined gravimetrically and by FTIR spectroscopy. The process was related to an expected stiffening of the material through DSC measurements, which showed a noteworthy increase in the glass-transition temperature of the polymer from the initial −50 °C to a value close to room temperature (Figure 5). Indeed, a partial decrease in the flexibility has already been macroscopically observed since the artwork fabrication in 1999 and is considered the reason for the observed easier breakage of the thinner pieces of the installation.

### 3.2. Oxidation and Hydrolytic Degradation

**Figure 6:**
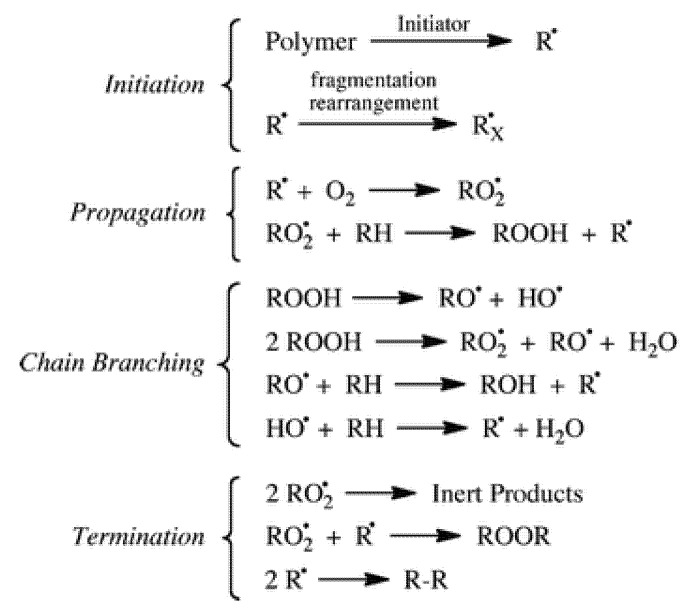
Simplified autoxidation scheme. Reprinted with permission from [56].

Ageing of most artworks entirely or partially made of plastics is essentially due to oxidation through an auto-accelerating mechanism, where after a certain induction period with no significant changes, the degradation rate increases rapidly. Based on the well-known cycle of oxidation of hydrocarbons and, by extension, of polymers (simplified scheme in Figure 6) [55,56], the initiation step consists of the abstraction of some extremely labile hydrogen, followed by oxygen addition to form reactive peroxy radicals. The initial radical species are formed even under indoor environmental conditions by photo-transformation or thermal decomposition of photochromic or other unstable impurities or structural defects. After abstraction of labile hydrogens from the polymer backbone, unstable hydroperoxides are formed that further evolve in a secondary cycle, whereas the resulting polymeric radicals feed the propagation cycle.

From the evolution of the hydroperoxides to alkoxy radicals and further chain branching reactions (not detailed in the scheme), a series of oxygen-containing species such as ketones, aldehydes, acids, esters and alcohols are formed. Such degradation products have functional groups that were not present in the pristine material; they may be chromophores and eventually have molecular weights far different from the initial ones due to either secondary cross-linking or scission reactions. Fragments may have molecular weight down to some thousands of Da (i.e., the so-called low molecular weight degradation products) or even be volatile (usually known as volatile organic compounds, VOCs).

If oxidation is especially important for vinyl polymers, the specific structural features of condensation polymers also make them suitable for hydrolysis, usually as a first degradation step. The hydrolytic degradation consists in the reaction of polymers with water, resulting in the cleavage of susceptible functional groups, commonly into chemical species that resemble the monomers used in the polycondensation synthesis. Polymers inherently susceptible to hydrolysis are those forming water during polymerisation, such as UP, PA and PUR, and also semi-synthetic polymers such as cellulose esters. To a lesser extent, ER and other thermosetting resins are also exposed to hydrolytic degradation.

The formation of new functional groups as an effect of oxidation or hydrolysis is limited by oxygen or water diffusion, respectively, to the surface of the artworks and may be easily detected by direct methods such as different vibrational spectroscopy techniques, or indirectly through, e.g., colour detection, UV-vis or fluorescence spectroscopy [57], in some cases supported by more time-consuming and sophisticated techniques (NMR, GC/MS, etc.). On the other hand, molecular weight changes resulting from oxidation or hydrolysis processes are detectable by size exclusion chromatography (SEC) and gravimetric methods through insoluble determination. In contrast, the evolution of VOCs may be quantified by weight loss measurements and measured by different GC/MS methodologies [58].

**Figure 7:**
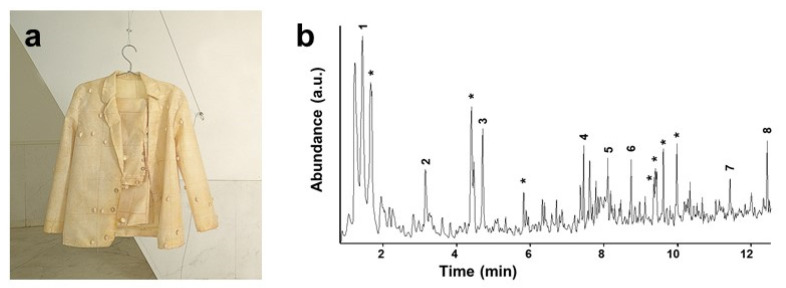
“Traxe de home” (1996) by A. Pinal (**a**) and py-GC/MS chromatogram of the latex textile (direct pyrolysis at 600 °C) (**b**). MS identified the peaks as: isoprene (**1**), toluene (**2**), xylene (**3**), dimeric isoprenes (**4**–**6**) and trimeric isoprenes (**7**,**8**). Asterisks denote aliphatic or aromatic alcohols, aldehydes and ketones. Image reprinted with permission from [30].

A representative example of applying basic analytical techniques to oxidation studies concerns the monitoring of artworks made of easily oxidisable polydiene elastomers, with natural PI being the most commonly used. The faster than expected [59] deterioration of a latex suit made by A. Pinal, shown in Figure 7, could be pointed out by ATR-FTIR data collected from some detached fragments [30]. Colour evolution and an increase in stiffness have been related to the appearance of the typical products of hydrocarbon oxidation, visible as a broad stretching vibration band of hydroxyl groups at 3550–3100 cm^−1^ and carbonyl absorption centered around 1720 cm^−1^ due to ketones, with shoulders at lower and higher wavenumbers. Additional information on the conservation state of the artwork was also revealed by py-GC/MS analysis, also shown in Figure 7; apart from the obvious identification of isoprene and isoprene oligomers, the abundant presence of oxygen-containing compounds such as aliphatic and aromatic alcohols, aldehydes and ketones was related to the pyrolytic decomposition of strongly oxidised PI molecules.

**Figure 8:**
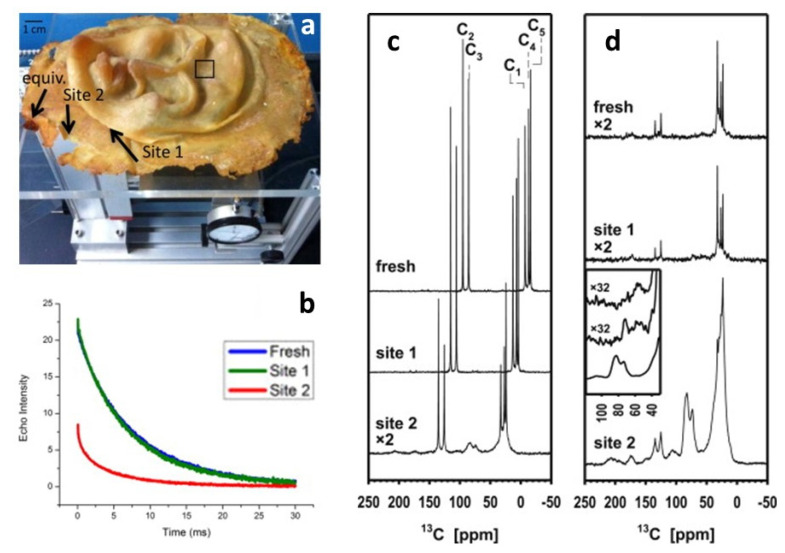
(**a**) Five-year-old natural latex mask by L. Isolani on unilateral NMR instrument. Arrows mark the less degraded site 1, site 2 and a site with degradation similar to site 2. (**b**) Relaxation decays acquired with unilateral NMR (blue: fresh reference sample; green: site 1; red: site 2). (**c**) ^13^C DEPTH spectra of a fresh preparation of natural rubber and samples of the artwork taken from site 1 and site 2. The upper two spectra are shifted for better visibility. (**d**) ^13^C CP spectra of the same samples. The insert shows excerpts of the CP spectra with more line broadening (100 instead of 20 Hz; the order is top: fresh, center: site 1, bottom: site 2). Image reprinted with permission from [60].

The effects of the oxidation on the same polymer but in another work of art were also investigated by two types of NMR [60], performed at two sites that had been identified as extreme cases (as shown in Figure 8, site 1 does not show signs of deterioration, while site 2 is visually degraded). Noninvasive unilateral NMR easily differentiated between them and determined the depth to which the material is affected, resulting in fast signal decay due to low molecular mobility where the material has lost elasticity. However, micro-destructive analysis by ^13^C solid state NMR added structural details. Both ^13^C distortionless enhancement by polarisation transfer (DEPTH) and especially cross-polarisation experiments, where the signals of the rigid components are amplified from the protons, show the presence of the aliphatic polyisoprene signals and many new peaks, indicating that there are several processes of chemical alteration responsible for the apparent degradation. New signals were identified as follows: (i) a minor signal peaking at 208 ppm corresponding to ketones and aldehydes, usually considered the product of the main product of the dominating process; (ii) a signal at 178 ppm corresponding to carboxylic acids; (iii) a signal at 105 ppm due to the formation of new double-bonded carbons; (iv) the dominating signal at 83 ppm with a side peak at 73 ppm, that constitutes 15% of all carbon in the quantitative DEPTH spectrum, corresponding to carbons with a single bond to oxygen, possibly generated through the formation of oxygen cross-links that would explain the hardening of the material.

**Figure 9:**
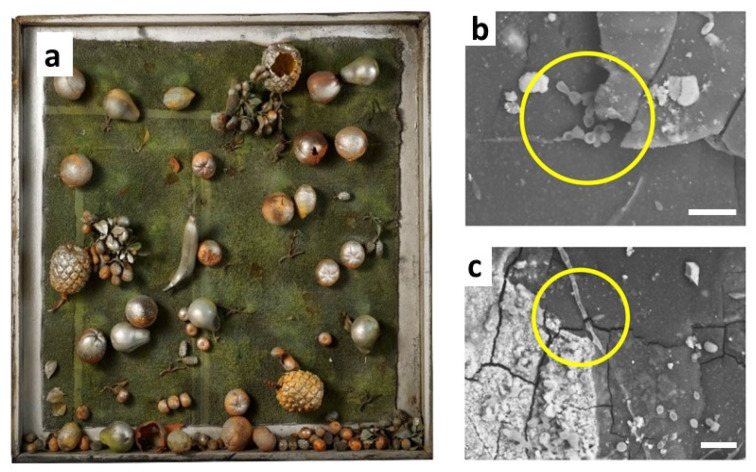
“Teca con frutta” (1967) by M. Zuppelli (**a**) and SEM images of samples from the surface of PE fruits (**b**,**c**). Scale bar 20 mm. Yellow circles highlight the presence of hyphae and spores. Image reprinted with permission from [61].

Another family of vinyl polymers, the so-called polyolefins, essentially consisting of PE and PP, are the leading industrial polymers for a wide range of commercial applications but were only rarely used by artists. Recently, the limited stability of a 50-year-old artwork partially made of low-density PE has been revealed by micro-FTIR analysis on diamond cells [61]. Samples as small as a few micrometres showed the formation of multiple auto-oxidation products, such as ketones, esters and γ-lactones. In the end, this micro-destructive technique also enabled the detection of products of biological degradation, and in particular oxalic acid salts, possibly resulting from the attack of fungi on the nitrocellulose surface coating. The hypothesis of a rather uncommon biological colonisation was confirmed by direct visualisation of spores (rounded reproductive structures) and hyphae (elongated filamentous elements) by SEM (Figure 9).

Among polycondensation polymers, it is well known that PUR, especially in the form of foams, may deteriorate in a few years as an effect of moisture and light, showing discolouration, loss of flexibility and crumbling. Studies on materials for industrial purposes demonstrate that poly(ester urethane) is more sensitive to hydrolysis while poly(ether urethane) is more prone to oxidation [62,63]. Concerning their use in artworks, investigations for the conservation of Gilardi’s sculptures made of poly(ester urethane) foam easily detected the chemical changes taking place during deterioration by ATR-FTIR [42] and micro-FTIR [64]. Such modifications essentially correspond to an increase in the absorption of hydroxyl groups and changes in the carbonyl region. Furthermore, the level of degradation of PUR foams of diverse artworks has been quantitatively evaluated by calculating the ratio between a reference band of either the ester or the ether and the absorption of the hydroxyl group [36,42]. Micro-FTIR spectroscopy also enabled the detection of the extensive ageing of PI latex used by Gilardi to protect PUR ether foams, possibly accelerating its degradation [64].

More detailed studies on the complex mechanism of degradation of PUR foams, consisting of parallel processes of photolysis, oxidation and hydrolysis, together with cross-linking of the hydrolysed fractions, required the use of techniques that enable the analysis of microstructures. Py-GC/MS in combination with EGA-MS of the material used in “Contenitoreumano” and “Moon Surface” by Turcato enabled not only the identification of the components discussed in the previous section but also suggested a change in the structure of the polymer as a result of 50 years of ageing [43,44]. Similarly, the previously mentioned application of liquid and solid state NMR for the identification of the PUR foam used in “Moon Surface”, also found that the degradation processes mostly affected the ethylene units capping polyether segments of the foam [45].

A different methodological approach that is worth discussing in detail is that applied to follow the deterioration due to hydrolysis in cellulose plastics, specifically tested in CN and CA sculptures realised by N. Gabo and A. Pevsner, and L. Moholy-Nagy and M. Duchamp, respectively [65], to address conservation treatments. Previous studies showed that early signs of hydrolysis of cellulose esters might be detected by FTIR spectroscopy [66] and were revealed more in detail by py-GC/MS [67]. In highly degraded CA, the loss of characteristic spectral features associated with the acetate group made micro-FTIR analysis inconclusive. Only py-GC/MS was shown to be a specific and sensitive technique for the identification of deacetylated CA, based on the detection of trace levels of characteristic marker compounds. On the other hand, quantitative evaluation of the level of deterioration of cellulose plastics from different sculptures or different parts of the same sculpture required liquid chromatography techniques. Degradation essentially consists of a combination of two different progressive processes: (i) loss of acetates or nitrates from the cellulose rings, which could be quantified by an ion chromatography method capable of analysing anions and cations, such as acetic acid or nitrite and nitrate from cellulose acetate and cellulose nitrate, respectively; (ii) reduction of the molecular weight due to chain breaking, followed by SEC. As general conclusions, CA objects showed a degradation essentially consisting of an extensive acetic acid loss with a low molecular weight decrease. In contrast, CN showed a much larger molecular weight decrease [65].

In all the cases in which in situ surface analysis techniques or invasive sampling may not be performed, the identification of chain scission products considered as markers of degradation provided a new powerful diagnostic tool. Curran and colleagues introduced VOC analysis to classify polymeric museum artefacts according to their degradation [58]. Solid phase microextraction gas chromatography mass spectrometry (SPME-GC/MS) was used to detect volatile products of degradation released from a series of 96 reference polymeric museum objects dating from the 1920s–2000s. Identification of VOCs gave insight into the composition and ongoing degradation of the objects made of CA, CN, PS and some PUR and, in contrast, classification of PE and PVC samples was poor. On such a basis, the method was successfully applied to three modern artworks by Naum Gabo and Antoine Pevsner made of CA and CN, thus showing their different levels of degradation [25,58].

More recently, the combination of a nano-destructive sampling procedure with the surface enhanced Raman spectroscopy (SERS) sensitivity allowed for achieving the detection of non-volatile low molecular weight degradation products formed at the surface of reference polymers and was applied to follow the deterioration of PI-made artworks [68]. Silicon sampling strips enable the efficient extraction of oligomeric degradation markers for amounts as small as 10^−10^ g, whereas the use of novel 3D SERS substrates [69] provides their direct identification, even in conditions in which traditional spectroscopic techniques or py-GC/MS do not have enough sensitivity.

### 3.3. Simulation of Ageing to Predict Artwork Durability

In addition to assessing the current state of the conservation of artworks, possibly through the detection of the effects of degradation on the constituent materials, the prediction of their long-term behaviour plays an important role to ultimately address conservation and management strategy. It is usually assumed that the polymeric parts of museum objects are the more degradable interface with the environment and that their overall durability is directly related to the ageing of such critical components. A common material science practice to investigate polymers’ weathering, in times shorter than those necessary under natural conditions, consists in the exposure of reference samples to simulated oxidative conditions [70]. Ageing may be accelerated through two main approaches: irradiation of the polymer samples in a medium-accelerated photo-ageing device equipped with a solar-like lamp (filtered for ≤ 295 nm), or an isothermal treatment in a forced-air circulation oven at a temperature lower than the temperature of glass transition, under controlled conditions of humidity. To dispel misunderstanding, it is necessary to point out that the conditions of simulated ageing assays do not trigger unexpected reactions: either artificial solar light or isothermal treatment at a moderate temperature only accelerate the same chemical changes as those occurring in the long term under the permanent physico-chemical stresses of the environment [3,70], including those found in museums. Isothermal treatments are also used to simulate the migration of additives from polymeric materials, as shown in Section 3.1. In this case, the increase in temperature has the function of accelerating a physical process, i.e., the evaporation of low molecular weight organic molecules, instead of the kinetics of chemical reactions.

Specimens for the assays may be:

- sampled from the artwork;
- supplied directly by the artist as material remainders available in their studio;
- prepared from the commercial materials stated by the artist;
- selected among commercial materials similar to the one used in the artwork.

The effect of the accelerated ageing on polymer structure and molecular weight may be followed over the treatment by any spectroscopic, chromatographic or thermal analysis techniques, including those that are not usually applied to artworks due to limitations in terms of sample amount or surface area. In addition, the evolution of volatiles, weight and surface changes may also be monitored to follow the progress of degradation reactions.

**Figure 10:**
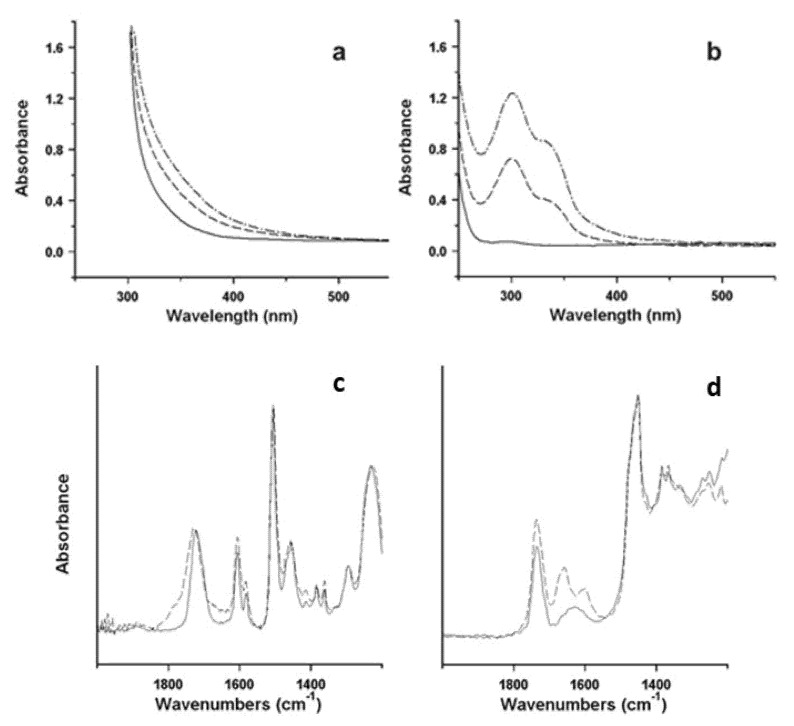
UV-vis spectra of reference films of: (**a**) the epoxy vinyl ester resin before (solid line) and after 24 (dashed line) and 120 h (dash-dotted line); (**b**) the aliphatic ER before (solid line) and after 100 h (dashed line) and 200 h (dash-dotted line) accelerated photo-ageing. ATR-FTIR spectra between 2000 and 1250 cm^−1^ of: (**c**) the epoxy vinyl ester resin before (solid line) and after 1000 h (dashed line) isothermal treatment; (**d**) the aliphatic ER before (solid line) and after 200 h (dashed line) photo-ageing. Image reprinted with permission from [71].

Illustrative examples of the procedure are represented by two studies of the degradative behaviour of different types of ER, namely, an aliphatic ER and an epoxy vinyl ester, used for surface finishing of sculptures realised in 2003 and 1998, respectively [27,30,71]. Reference samples in the form of thin films, prepared from the commercial materials used by the artists, have been submitted to artificial ageing, and changes induced by degradation have been periodically checked. A progressive yellowing has been observed and better quantified by UV-vis spectra, as shown in Figure 10a,b, where the increase in absorption in the aliphatic ER could be attributed to the development of specific chromophore groups, possibly quinoid structures. FTIR spectra (Figure 10c,d) unveiled the formation of amides in the aliphatic ER. The epoxy vinyl ester resin mainly showed a progressive broadening of the carbonyl ester absorption, and a limited increase in the broad OH stretching band (not visible in the figure), both due to the formation of new oxidised structures, which are possibly related to the chromophores responsible for yellowing. Based on the evaluation of the results from artificial ageing and remembering that the understanding of the degradation process is crucial for predicting the artworks’ durability, it is usually possible to speculate on the mechanism of oxidation.

**Figure 11:**
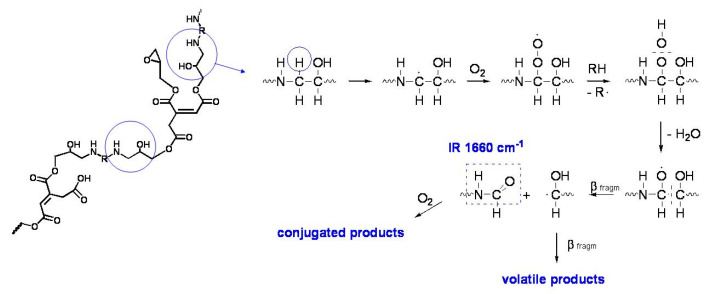
Simplified mechanism of oxidation of the aliphatic ER. The structure of the cured epoxy network is proposed based on the composition of the epoxy prepolymer and the polyamine hardener used for its preparation. Image reprinted with permission from [30].

**Figure 12:**
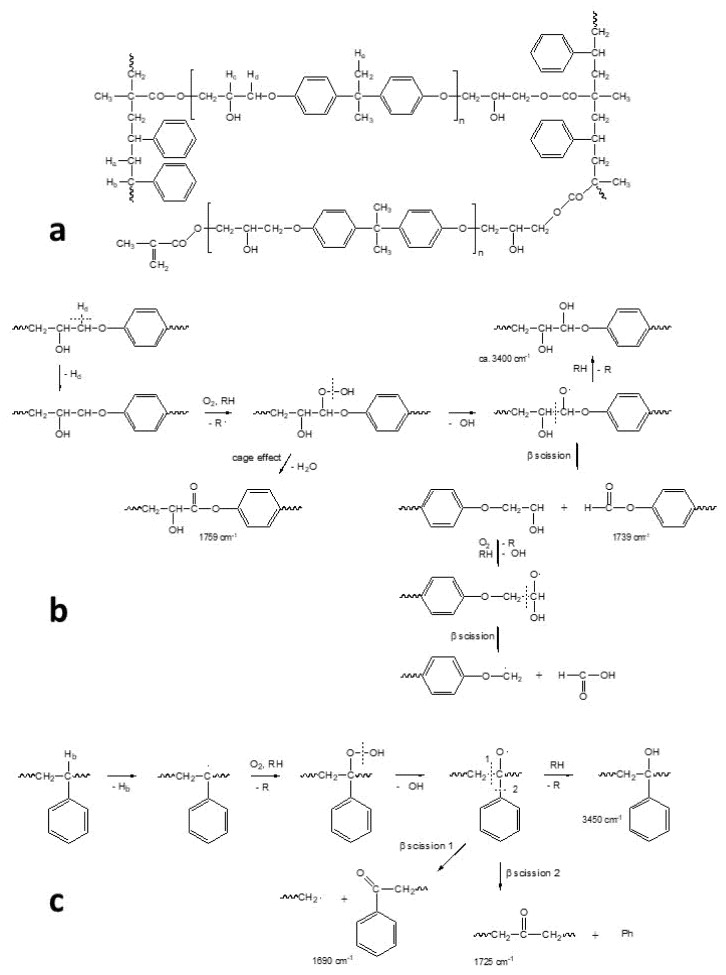
Schematic structure of the epoxy vinyl ester network showing the main different types of hydrogen atoms (**a**). Schemes of the main oxidation reactions taking place in the epoxy vinyl ester prepolymer residues (**b**) and in the styrene residues (**c**), as well as the absorption peaks in the infrared region of the main products. Image reprinted with permission from [27].

In the specific case of the ER, it has been supposed that the degradation takes place through the typical auto-oxidation process. The initial step consists of the abstraction of some labile hydrogen, e.g., the one adjacent to the amine group in the aliphatic ER (Figure 11), and the hydrogen in the α position to the oxygen of the ether group and the tertiary hydrogen in the styrenic units in the epoxy vinyl ester resin, followed by oxygen addition and the formation of hydroperoxide intermediates (Figure 12). For the aliphatic ER, such intermediates may evolve into amide groups. In contrast, in the epoxy vinyl ester resin, the large variety of labile hydrogens easily cause the formation of oxygen-containing groups with different local structures, responsible for the increase in carbonyl and hydroxyl absorption in the FTIR spectra. Such mechanisms also justify the formation of the small amounts of volatile compounds revealed by gravimetric measurements.

**Figure 13:**
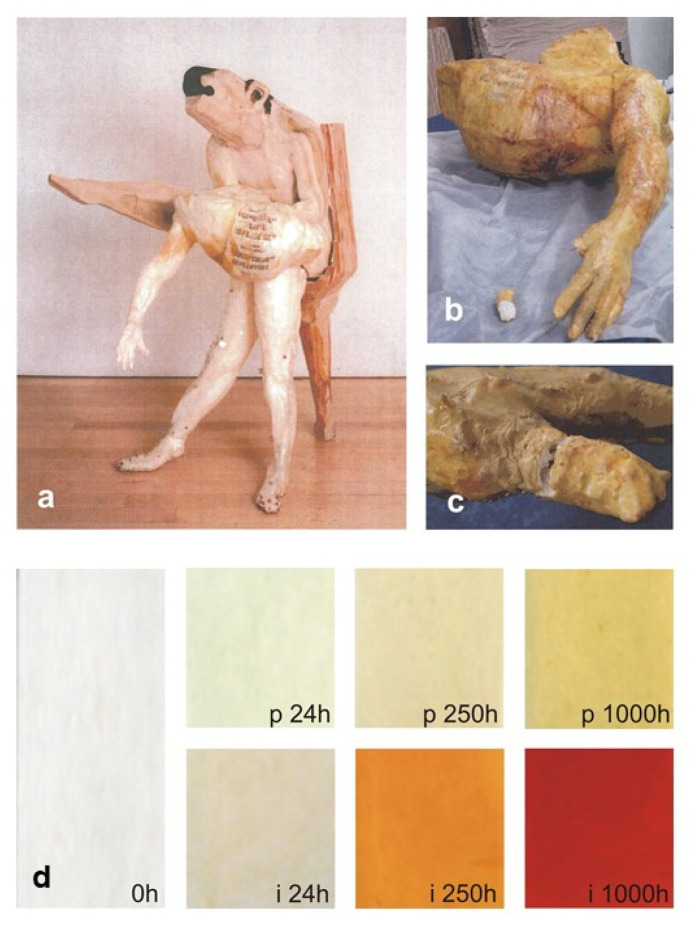
Comparison between “Nemeas Lion” (1998) by F. Leiro (**a**), pictures taken in 2012 of part of the human body (**b**) and a detail of the hand (**c**), and artificially aged reference epoxy vinyl ester films (**d**) before (labelled as 0h) and after 24, 250 and 1000 h of photodegradation (p24h, p250h and p1000h) or isothermal (i24h, i250h and i1000h) treatments. Image reprinted with permission from [27].

Phenomenological and mechanistic aspects concerning the behavior of the critical polymeric component could be corroborated through a comparison with the results from the evaluation of the actual state of the conservation of sculptures. ATR-FTIR spectroscopy alone provided a clear correspondence between artificial and natural ageing of both ERs, and also the formation of chromophores has been progressing quickly in a museum environment, as predicted. For example, the yellowing due to artificial photo-ageing of the epoxy vinyl ester is compared in Figure 13 with that of a sculpture after 14 years of natural ageing.

In addition, other predictions on the durability of the following reference polymers could be compared with the natural ageing behaviour of other artworks: PI (latex suits realised in 1999) [30], poly(ester urethane foam) (Gilardi’s sculptures, 1960–1990) [42], poly(ether urethane elastomer) (L. Cecchini, 1999) [68], poly(ether urethane) foam [42], PA (Esferobite DSk, 2008) [50], UP resins (different artists, 1987 and 1992) [48]. There was always a general good agreement between simulated and natural ageing, confirming the potential and versatility of the approach.

As a final remark in this section, it must be stressed that a simulated ageing treatment does not predict in how many years the polymeric material, and by extension, the artwork, will deteriorate but only give an idea about its weatherability and, in some cases, enables comparing its stability with that of other reference materials. In fact, it is well known that the accelerating factor of the selected artificial ageing test may be only estimated from actual use of the specific material, and the correlation is always approximate since under natural exposure, the ageing is influenced by a series of factors which are of difficult reproducibility in accelerated tests [70,72].

## 4. Summary and Outlook

An overview of the studies devoted to the assessment of contemporary artworks partially or entirely made of plastic shows that vibration spectroscopies, and especially ATR-FTIR spectroscopy, are the appropriate techniques to identify polymeric material constituents, and may also detect signs of ageing, either due to additive migration, oxidation or hydrolysis. Whenever micro-sampling is not possible or discouraged, the recent availability of several portable systems (e.g., portable FTIR reflectance spectroscopy) offers an alternative, often unique, option to enable identification. The further development and broader application of instruments actually in use for other cultural heritage objects and barely experimented on plastic artworks, such as handheld Raman spectroscopy [73], in situ diffuse reflection infrared Fourier transform (DRIFT) spectroscopy [74] or fibre optic Fourier transform mid-IR reflectance spectroscopy (FORS) [32,75], will allow systematic detection campaigns on museum artefacts.

When IR analysis does not unequivocally identify polymer structures (as in the case of many polycondensation polymers, e.g., PURs and polyesters) or functional groups resulting from degradation processes, other techniques must be applied, alone or in combination with vibrational spectroscopies. Py-GC/MS not only revealed the composition of different types of PUR foams, polyesters and many polyaddition polymers used by diverse artists over recent decades but also allowed the evaluation of the ageing of, e.g., PUR and PI artworks. NMR spectroscopy is a potentially excellent technique to detect structural changes in polymers and may be used as a diagnostic alternative to follow any type of degradation but has the limitation of requiring larger amounts of sample than py-GC/MS. Notwithstanding, several different NMR experiments already showed their potential for polymer oxidation studies, with a special mention for the first application of a noninvasive tool, i.e., unilateral NMR, to follow chemical processes in situ, through direct analysis of artworks. Changes of T_g_ (usually determined by DSC) and weight loss are both excellent indicators to track the loss of plasticisers, while contact angle measurements are the best choice to follow the blooming of higher molecular weight additives. Visual inspection, UV-vis spectroscopy or colour measurements (typically by a portable spectrophotometer) should also always be carried out to follow surface deterioration, as colour changes due to the formation of very small amounts of chromophores must be considered as early markers of polymer degradation, and enable the investigation of oxidation/hydrolysis kinetics.

All these methods disclose the structural and molecular changes taking place due to ageing and permit the proposal of detailed degradation mechanisms. This knowledge, resulting from either the evaluation of the current state of conservation or through simulation tests of model polymers, is essential to predict further ageing and durability of artworks.

With respect to future trends, further efforts are expected in the fine-tuning of the analytical procedures aiming to detect the low molecular weight products of degradation of artwork plastics, either in the form of VOCs or released as oligomeric compounds onto the surface. The development of easy-to-use museum kits to sample on site and instrumentally detect such products may be considered as the final objective of an innovative strategy to diagnose surface degradation in polymeric museum artefacts. In addition, the application of a reflectance hyperspectral imaging technique initially introduced to perform, at once, noninvasive diagnostics, analysis and documentation of artworks such as paintings and polychrome surfaces [76,77] is also expected to be expanded to the study contemporary artworks, although with the current limitation of 2D applicability.

## Figures and Tables

**Table 1:** Synthetic polymers used in contemporary art sculptures and installations.

Polymers ^1^	Artist ^2^
HDPE	Massimo Zuppelli
PP	Pino Pascali
PI	Andres Pinal, Eva Hesse
PMMA	Angelo de Sousa, McCracken, Larry Bell, Robin Irwin, Fred Dreher, William Reimann, Ted Hallman, Freda Koblick, Donald Judd, Jesús Rafael Soto, Fernandez Arman, Naum Gabo, Vasa Velizar Mihich
PS, ABS	Joseph Konzal, Claudia Hart, Erutti
plast. PVC	Alberto Burri, Christo and Jeanne Claude, Duck-Bong Kang
PUR	Pietro Gilardi, Lorenzo Quinn, Gaetano Pesce, Thelma Newman, Loris Cecchini, Ted Hallman, Giulio Turcato, Sante Monachesi, Urs Fischer
PA	Naum Gabo
ER	Karin Sander, Edward Higgings, Francois Xavier Lalanne
MF	Sergio Lombardo
UP	Alberto Burri, Cracking Art Collective, Tony Cragg, Jess Koon, Niki de Saint Phalle, Naum Gabo, Robert Mallary, Yves Klein, Thelma Newman, John Luis de Andrea, Etienne Bossut, Martine Orsoni, Francois Xavier Lalanne, César, Maurizio Cattelan, Erutti

^1^ HDPE: high-density polyethylene; PP: polypropylene; PI: polyisoprene; PMMA: poly(methyl methacrylate); PS: polystyrene; ABS: acrylonitrile butadiene styrene copolymer; plast. PVC: plasticised poly(vinyl chloride); PUR: polyurethane; PA: polyamide; ER: epoxy resin; MF: melamine-formaldehyde resin; UP: unsaturated polyester resin. ^2^ From catalogues of collections of museums, auction galleries and specialised libraries: plastic.syr.edu, artprice.net, artsy.net, saatchiart.com, Sotherby’s, Christie’s, Judd Foundation, americanswedish.org, Archive of American Art, the Museum of Modern Art, Guggenheim Museum, Tate Modern Gallery, Los Angeles County Museum of Art, Museo d’Arte Contemporanea Roma.

**Table 2:** Techniques already applied for the identification and compositional analysis of plastics in contemporary artworks, listed by polymer family.

Techniques	Polymers ^1^											
	PE,PP	PI	PS	PMMA	PET	PVC	PUR	PA	ER	UP	CA,CN	Others
(ATR) FTIR ^2^	◯	◯	◯	◯	◯	◯	◯	◯	◯	◯	◯	◯
Other IR/Raman ^3^	◯	-	-	◯	-	-	-	-	-	-	◯	◯
Py-GC/MS	-	☐	◯	◯	◯	-	☐	☐	☐	☐	◯	◯,☐
NMR ^4^	-	-	-	-	-	-	☐	-	-	☐	-	
DSC	-	-	-	-	-	-	-	☐	-	-	-	-
EDXRF	◯	-		◯	◯	◯	-	-	-	-	-	-

^1^ Symbol ◯ refers to: general identification of the polymer family; ☐: specific structural analysis within the family; ^2^ including micro-FTIR; ^3^ including reflectance mid-FTIR spectroscopy, Raman spectroscopies; ^4^ including 1D and 2D NMR experiments, either in solution or in the solid state.

## Data Availability

Not applicable.
